# Effects of Contemporary Irrigant Activation Schemes and Subsequent Placement of an Interim Dressing on Bacterial Presence and Activity in Root Canals Associated with Asymptomatic Apical Periodontitis

**DOI:** 10.3390/jcm9030854

**Published:** 2020-03-20

**Authors:** Alexandre P. L. Carvalho, Laura C. L. Nardello, Fernanda S. Fernandes, Fernanda P. Bruno, Luiza R. Paz, Elaine F. Iglecias, Heitor M. Honório, Márcia P. A. Mayer, Giulio Gavini, Ericka T. Pinheiro

**Affiliations:** 1 Department of Dentistry, School of Dentistry, University of São Paulo, São Paulo 05508-000, Brazil; alelimacarvalho@usp.br (A.P.L.C.); laura.nardello@usp.br (L.C.L.N.); fernanda-fernandes@usp.br (F.S.F.); fernanda.bruno@usp.br (F.P.B.); luizarp@usp.br (L.R.P.); efiglecias@usp.br (E.F.I.); ggavini@usp.br (G.G.); 2 Department of Pediatric Dentistry, Orthodontics and Public Health, Bauru School of Dentistry, University of São Paulo, Bauru 17012-901, Brazil; heitorhonorio@usp.br; 3 Department of Microbiology, Institute of Biomedical Sciences, University of São Paulo, São Paulo 05508-000, Brazil; mpamayer@icb.usp.br; 4 Clinic of Conservative and Preventive Dentistry, Center of Dental Medicine, University of Zurich, CH-8032 Zurich, Switzerland

**Keywords:** root canal treatment, endodontic instruments, endodontic irrigation, calcium hydroxide, quantitative polymerase chain reaction

## Abstract

New tools for activating endodontic irrigants have evolved, yet their impact on root canal disinfection, in comparison to the passive placing of an inter-visit medication, have not yet been fully elucidated. The use of DNA- and rRNA-based methods may cast some new light on this issue, as they allow a comparison to be made between microbial presence and activity. Therefore, the aim of this single-arm intervention trial is to evaluate the antibacterial effect of endodontic procedures using both molecular methods. Root canal samples were obtained from 20 patients with asymptomatic apical periodontitis after each treatment step: access cavity, chemo-mechanical preparation, adjunctive procedures (XP-endo Finisher file and passive ultrasonic irrigation), calcium hydroxide medication, and 2nd-visit root canal preparation. DNA and cDNA from the samples were subjected to quantitative polymerase chain reaction with universal primers for the bacterial 16S rRNA gene. Chemo-mechanical preparation promoted a drastic reduction in bacterial levels and activity, whereas the adjunctive procedures did not make a significant contribution to further disinfection. At the 2nd visit, bacteria were active after the use of calcium hydroxide medication; however, they were significantly reduced after a 2nd-visit preparation. Consequently, the lowest bacterial levels were found at the end of the treatment. This clinical trial, which used an rRNA and rDNA combined approach, confirmed previous studies showing that root canal preparation represents the main strategy for root canal disinfection.

## 1. Introduction

The aim of endodontic treatment of teeth with apical periodontitis is to reduce viable bacteria to levels that are compatible with apical healing. Culture-dependent techniques have been used extensively for counting viable bacteria after endodontic procedures [1]. However, since an endodontic microbial community comprises many as-yet-uncultivated/difficult-to-culture bacteria [2], molecular methods that target viable cells [3] or culturomic analysis [4] may be useful strategies for monitoring bacterial load during endodontic treatment.

Most molecular-based studies have used assays that target bacterial ribosomal RNA genes (rDNA), usually the 16S rRNA genes [4]. Although rDNA-based quantitative polymerase chain reaction (qPCR) may provide important information on the bacterial load remaining after treatment, they are not suitable for assessing bacterial viability [5]. However, qPCR assays that target bacterial ribosomal RNA (rRNA) could be useful for this purpose [6]. As rRNA levels are related to the bacterial activity, the abundance of rRNA transcripts in relation to their corresponding genes (rDNA) has been used to search for active bacterial cells in microbial communities [3,7,8,9,10].

Despite the drastic reduction in bacterial levels, several canals remain infected after chemo-mechanical preparation (CMP) [11,12,13]. Therefore, supplementary approaches have been recommended for improving root canal disinfection following CMP [14]. Approaches for supplementing the disinfection at the first treatment visit are mainly based on irrigant agitation techniques, which are used after CMP. Fluid movements or mechanical forces generated by different devices, aim to remove bacteria and debris from untouched areas of the root canal [14]. Passive ultrasonic irrigation (PUI) has frequently been used for this purpose as it promotes acoustic streaming and cavitation forces in the irrigant [15,16]. More recently, XP-endo Finisher (XPF) (FKG Dentaire, La Chaux-de-Fonds, Switzerland) has been introduced as a new adjunctive approach. The instrument’s properties allow better contact between both the instrument and the irrigant with the dentin walls [17]. Laboratory studies using XPF as an adjunctive procedure after CPM have shown promising results [18,19]. However, its clinical impact on the root canal disinfection process has yet to be proven.

The use of an inter-appointment medication to maximize root canal disinfection is one of the most controversial issues in endodontics [20]. Molecular-based clinical studies to evaluate the antimicrobial effectiveness of calcium hydroxide as an intracanal medicament have shown divergent results. Some authors have reported reduced bacterial levels after inter-appointment medication in comparison to first visit treatment procedures [21,22], whereas others found no difference [23,24], or even an increase in rDNA levels following Ca(OH)_2_ use [25].

Although some researchers have claimed that using supplementary procedures after CMP at first treatment visit has the potential to eliminate second visit antimicrobial strategies, microbiological clinical studies to validate both treatment regimens are still needed [14]. Comparing rDNA and rRNA may cast some new light on this issue, as these methods allow bacterial levels and activity to be compared. Therefore, this study aims to use molecular methods to assess the viable bacterial load in root canals during a treatment protocol that uses a new tool to activate endodontic irrigants after CMP and a subsequent two-visit antibacterial approach.

## 2. Materials and Methods

### 2.1. Patient Selection

The study was conducted in accordance with the Declaration of Helsinki, and the research protocol was approved by the Institutional Ethical Committee (#2.201.768, 04/08/2017). All the selected patients gave signed informed consent before the treatment. Inclusion criteria were patients with single- rooted teeth and asymptomatic apical periodontitis. Exclusion criteria were patients who had received antibiotic therapy during the previous 3 months or had general disease, teeth that could not be properly isolated with a rubber dam, non-restored teeth, periodontal pockets depths greater than 4 mm, and radiographic evidence of previous root canal filling, open apex, crown/ root fracture, root resorption, or narrow canals.

### 2.2. Pilot Study

The treatment protocol for using XPF as an adjunctive procedure after CMP at the first treatment visit was determined based on a pilot study. The first 10 patients with single-rooted teeth and asymptomatic apical periodontitis were selected and the root canal samples were analyzed by an rDNA-based qPCR using universal primers for the bacterial 16S rRNA gene. Initially, XPF activation was performed using the parameters described in a previous study [22]: 30 s of activation with 2 mL of 2.5% NaOCl (2x), followed by 30 s of activation with 2 mL of 17% EDTA (2x), and 30 s of activation with 2 mL of 2.5% NaOCl (2x). However, a slight increase was observed in the bacterial load after XPF use (median 1.55 × 10^3^, range 0–5.64 × 10^4^), compared to the bacterial levels after chemo-mechanical preparation (median 9.75 × 10^2^, range 0–9.40 × 10^3^). Therefore, strategies to improve root canal cleaning were added to the final protocol; these included an irrigation step immediately after each XPF activation cycle and a final irrigation step using ultrasonic activation.

The endodontic treatment was performed over 2 visits, together with an inter-appointment medication and a second-visit root canal preparation. Based on the pilot study, the Wilcoxon test (power of 80%, significance level of 5%, and bilateral analysis) was used to assess differences in the bacterial levels between the first (median 1.55 × 10^3^, range 0–5.64 × 10^4^) and second visit endpoints (median 0, range 0–1.16 × 10^4^). A sample size of 17 patients was suggested, but it was decided to use a final sample size of 20 patients, due to the possibility of dropouts.

### 2.3. Clinical Study Design

**Figure 1:**
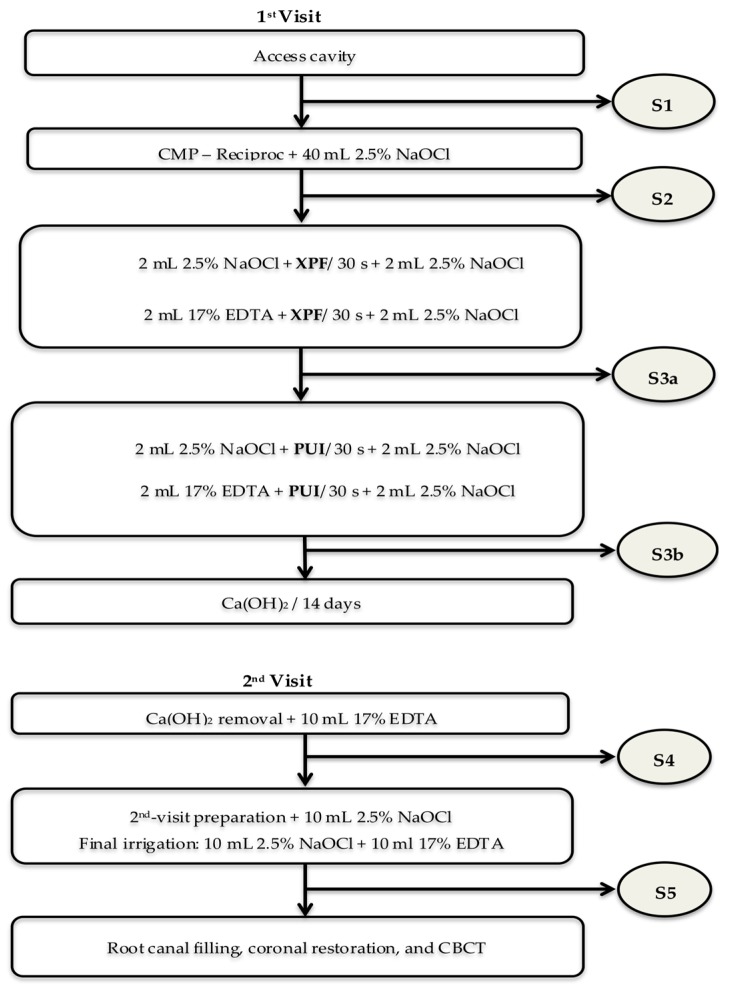
Flow chart of the interventions. The root canal samples were taken after access cavity (S1), root canal preparation (S2), XP-endo Finisher (XPF) (S3a), PUI (S3b), calcium hydroxide medication (S4) and 2nd-visit root canal preparation (S5).

This interventional study compared the antimicrobial effects of endodontic procedures (clinicaltrials.gov ID: NCT03537664). The interventions tested at the first and second visits are described in Figure 1. One endodontic specialist (APLC) who had limited his work to endodontics for the last 16 years, performed all the root canal treatments, whereas another investigator (LLN) performed the qPCR analysis.

### 2.4. Interventions and Microbiologic Samples

The microbiological sampling methods have been described previously [22]. First, tooth/rubber dam disinfection was performed using 30% H_2_O_2_ and 2.5% NaOCl for 30 s each, followed by 5% sodium thiosulfate (Na_2_S_2_O_3_). The access cavity disinfection was repeated before entering the pulp chamber, and a quality control (QC) sample was taken using sterile paper points. Then, the access cavity was completed using new sterile diamond burs irrigated with sterile saline. The paper points were placed in cryotubes containing 300 µL of RNAlater solution (Life Technologies, Carlsbad, CA, USA) and frozen at −80 °C for future DNA and RNA extraction. The absence of bacteria in the QC sample was verified by PCR and RT–PCR using universal primers for the domain *Bacteria*. If bacteria were detected in QC samples, the tooth was excluded from the study.

To obtain the initial root canal samples (S1), a #15 Hedström file was introduced into the canal, a filing motion was applied, and five sterile paper points ISO 20 (taper.02) were consecutively inserted to the working length (1 mm short of the apical foramen) for 1 min each. Then, the paper points and the file, without its handle, were transferred into cryotubes containing 300 µL of RNAlater solution, which were frozen at −80 °C.

Chemo-mechanical preparation (CMP) was performed using Reciproc NiTi instruments (VDW GmbH, Munich, Germany) according to the manufacturer’s instructions. Medium and wide canals were prepared with R40 and R50, respectively. After an initial flush with 10 mL of 2.5% NaOCl, the cervical, middle, and apical thirds of the root canal were sequentially prepared, with each step being followed by irrigation with 10 mL of 2.5% NaOCl. Consequently, a total of 40 mL of irrigant had been used by the end of root canal preparation. Then, the root canal was irrigated with 5 mL of 5% sodium thiosulfate and filled with sterile saline before the post-instrumentation sample was taken (S2).

The root canal was filled with 2 mL of 2.5% NaOCl and then activated for 30 s with the XPF instrument, which was connected to the VDW motor at 1000 rpm. The instrument was positioned 1 mm from the working length and gently moved up and down in the canal (7 to 8 mm-long movements). The XPF activation was followed by irrigation/aspiration with 2 mL of 2.5% NaOCl. Next, the remaining fluid was aspirated and 2 mL of 17% EDTA was inserted into the root canal and activated with XPF for 30 s, again followed by irrigation/aspiration with 2mL of 2.5% NaOCl. The canal was dried using paper points and flushed with 5 mL of 5% sodium thiosulfate for 1 min. The root canal was filled with sterile saline and a root canal sample was taken (S3a).

The PUI protocol following XPF activation was similar to that described above. Both 2.5% NaOCl and 17% EDTA were activated using a smooth wire with 0.2 mm diameter and 01 taper (Irrisonic- Helse, Ribeirão Preto, SP, Brazil), driven by an piezoelectric ultrasonic device (Piezo Light D5 Led, Olsen, SC, Brazil) set at 10% power in accordance with the manufacturer’s recommendations. The inserted tip was positioned 1 mm from the working length, avoiding contact with the root canal walls. Finally, 2.5% NaOCl was inactivated using 5% sodium thiosulfate and a new sample was taken at the end of the first visit (S3b).

UltraCal XS Calcium Hydroxide Paste (Ultradent Products Inc., South Jordan, UT, EUA) was used as an intracanal medication for 14 days. The paste was inserted into the canals using the 29G (0.33 mm) NaviTip (Ultradent Products Inc., South Jordan, UT, EUA) at the working length and packed to the level of the canal entrance. Radiographs were taken to check that the root canal was adequately filled with the intracanal medication. The access cavities were filled with 2 mm of temporary restorative material (Dentalvile, Joinville, SC, Brazil) and glass ionomer cement (Riva light cure, SDI limited, Bayswater, Victoria, Australia).

At the second visit, the operative field was disinfected, and a new quality control (QC) sample was taken. The intracanal medicament was removed using 10 mL of 17% EDTA and agitation with K-files, and a fourth sample was taken (S4). Next, the root canal was irrigated with 10 mL of 2.5% NaOCl and the second visit instrumentation was performed using the same Reciproc file selected for the first visit. A final irrigation was performed with 10 mL of 2.5% NaOCl, followed by 10 mL of 17% EDTA. A root canal sample was obtained at the end of the treatment, as described above (S5).

Root canal filling was performed, and the access cavity restored. An intraoral radiograph and cone beam computed tomography (CBCT) scans were taken to allow future analysis of the treatment outcome.

### 2.5. Nucleic Acids Extraction and cDNA Synthesis

DNA and RNA were extracted using the MasterPure Complete DNA and RNA Purification Kit (Epicentre Technologies, Madison, WI), as described previously [3]. Cell lysis was performed using 450 µL of tissue and cell lysis solution, 300 mg of glass beads (diameter, 0.1 mm; BioSpec Products Inc., Bartlesville, OK) and a Mini-Beadbeater (BioSpec Products Inc., Bartlesville, OK). Next, Proteinase K and MasterPure Complete protein precipitation reagent were added to the suspension sequentially, in accordance with the manufacturer’s instructions. Isopropanol was used to precipitate the nucleic acids, which were re-suspended in 35 µL of TE buffer.

The sample was divided in two vials: one was stored for further DNA analysis; and the other was subjected to RNA purification using the MasterPure Complete DNA and RNA Purification Kit (Epicentre Technologies, Madison, WI). The complementary DNA (cDNA) was synthesized using the SuperScript^®^ III First-Strand Synthesis System (Invitrogen) for reverse transcription (RT), in accordance with the manufacturer’s instructions. The DNA and cDNA were stored at −20 °C until use.

### 2.6. qPCR Assays

DNA and cDNA samples were used as templates for qPCR assays, which targeted conserved regions of the 16S rRNA gene of the *Bacteria* domain. The qPCR reactions (20 µL) contained 10 µL of Power SYBR Green Master Mix (Applied Biosystems, Foster City, CA, USA), 2 µL of template, and 100 nM of each primer (5′-CCA TGA AGT CGG AAT CGC TAG -3′ and 5′-GCT TGA CGG GCG GTG T-3′) [26]. The cycling conditions for qPCR reactions were 95 °C for 10 min, followed by 40 denaturation cycles at 95 °C for 15 s, and annealing at 60 °C for 1 min. The latter were performed using the Step One Plus thermocycler (Applied Biosystems, Foster City, CA, USA).

The exact number of total bacteria could not be calculated due to differences in the numbers of rRNA operons between bacterial species (ranging from 1 to 15). Therefore, bacterial levels were estimated based on a standard curve constructed with *E. faecalis*, which possesses 4 copies of rRNA operons. The standard curve was built using recombinant plasmids containing the 1500 fragments encoding the 16S rRNA gene of *E. faecalis* [3,22]. Plasmid standard dilutions (from 10^7^ to 10 DNA copies), DNA and cDNA samples were run in triplicate. The assay’s limit of quantification was 10^2^ DNA or cDNA copies; samples below the qPCR limit of quantification were considered negative. The ribosomal RNA gene (rDNA) levels were used for bacterial quantification, whereas ribosomal transcript (rRNA) levels were used to estimate bacterial activity at samples positive for rDNA.

### 2.7. Data Analyses

The nonparametric Wilcoxon signed rank test was used to determine differences in the bacterial levels before and after treatment procedures, and also differences between the rRNA and rDNA levels at each step of the treatment. Cochran’s Q test and the Wilcoxon signed rank test were used for qualitative analysis (incidence of qPCR positive samples before and after treatment procedures). McNemar’s test was used to compare the rRNA and rDNA-based qPCR assays’ detection rates. Differences were considered statistically significant if *p* < 0.05.

## 3. Results

**Figure 2:**
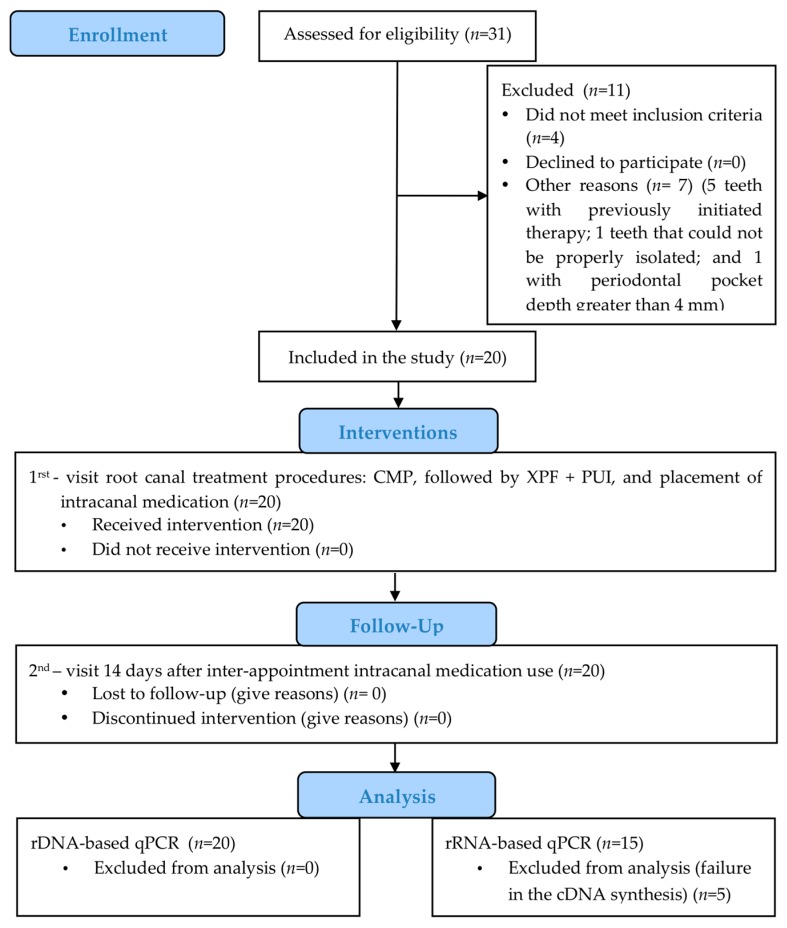
Flow diagram of the clinical trial. CMP, chemo-mechanical preparation; XPF, XP-endo Finisher; PUI, Passive ultrasonic irrigation; qPCR, quantitative polymerase chain reaction; rDNA, rRNA gene; rRNA, ribosomal rRNA.

Clinical characteristics of the participants are shown in Table 1. Five patients were excluded from the bacterial activity analysis due to the failure of the reverse transcription reaction (RT) after RNA extraction. A diagram illustrating the subject flow during the clinical trial is presented in Figure 2.

### 3.1. Bacterial Counts

All the cases were included for bacterial analysis because all the QC samples were negative. Quantitative and qualitative data for bacteria are summarized in Table 2. The rDNA-based qPCR assay revealed a significant reduction in the median number of bacterial cells in the S2 samples, compared to S1 (*p* < 0.0001). No significant decrease in bacterial counts was found when comparing S2 with S3a, S3b, or S4. However, the bacterial counts in S5 were significantly lower than in S2 (*p* = 0.0247), S3a (*p* = 0.0346), S3b (*p* = 0.0481), and S4 (*p* = 0.0031). In the qualitative (presence/absence) analysis, significant differences in the number of positive cases were observed between S1 and S2 (*p* = 0.0059) and S4 and S5 (*p* = 0.0207).

### 3.2. Bacterial Activity

The bacterial activity analysis was performed on root canal samples from 15 cases that were analyzed using both rDNA- and rRNA-based qPCR assays. The incidence of samples positive for bacterial rRNA and rDNA is shown in Table 3. Although the number of positive samples in the rRNA-based analysis was higher than in the rDNA-based, the difference in detection rates between the two methods was not statistically significant in most samples.

Table 4 shows the median rRNA and rDNA levels in samples positive for both assays. The concentration of rRNA copies was significantly higher than rDNA copies in the S1 samples (*p* = 0.0007) and S4 samples (*p* = 0.0499), indicating bacterial activity. No significant differences were found between the rRNA and rDNA levels in the S2, S3a, and S3b samples.

## 4. Discussion

This single-arm intervention trial compared the antimicrobial effects of endodontic procedures on the first and second treatment visits. The strengths of this study are the use of new tools for root canal disinfection, the strict inclusion criteria, and the use of a combined rRNA and rDNA approach for bacterial analysis. To the best of the authors’ knowledge, this is the first study to assess total bacteria activity in root canals by comparing rRNA and rDNA levels. The present study’s weaknesses include the small sample size and the analysis of only single rooted teeth. Difficulties imposed by the strict criteria used for patient selection in microbiological studies were the main reasons for the limited number of samples studied. Despite these limitations, the rDNA-based analysis of the 20 cases included in this study was sufficient to show differences in rDNA levels at the end of the first and second treatment visits (S3b and S5, respectively). The lowest bacterial rDNA levels were found at the end of the treatment, due to the cumulative effect of the first and second visit interventions, especially the chemo-mechanical preparation (CMP) and the second-visit root canal preparation. These findings reinforce the proposition that mechanical action with instruments and antimicrobial solutions are the main strategies for root canal disinfection [11,12,13,14,21,22,23,24,25].

In this study, the bacterial levels after CMP are in accordance with those reported in previous clinical trials using either reciprocating single instrument [22] or rotary multi-instrument systems [11,21,27]. The use of large volumes of irrigant and its frequent replacement may have contributed to the reduction in bacterial levels and activity in the present study [28]. Moreover, the depth to which the irrigating needle was inserted in the medium/wide canals studied may have been another factor contributing to the removal of bacteria from root canals [29]. However, despite the significant reduction in bacterial load after CMP, 60% of the cases were still positive for bacterial rDNA, which is in accordance with previous molecular-based studies [11,12,13,21,22,23,24,27].

The present study clinically assessed the antibacterial effect of XPF as an adjunctive procedure after CMP. Previous laboratory studies have recommended alternating cycles of XPF activation and irrigation for better biofilm removal [18]. The results of the pilot study confirmed this finding, as the bacterial levels tended to increase after XPF activation, probably due to the lack of proper irrigation between cycles. Therefore, additional irrigation volume and ultrasonic activation were implemented in the final protocol, with the aim of removing bacterial remnants suspended in the root canal lumen after XPF activation. However, even after the use of XPF and PUI for supplementary disinfection, 50% of the canals still had detectable levels of rDNA at the end of the first visit. Moreover, no significant difference was found in bacterial rDNA levels between the S2 and S3 samples. These findings are in line with a previous clinical study that evaluated PUI as adjunctive procedure [27]. However, it is important to note that the absence of a statistical difference between the S2 and S3 samples may be related to the small sample size used in the present study. Moreover, the inclusion of only single-rooted teeth may also have had an impact on the results, as XPF and PUI would be expected to be more beneficial in multi-rooted teeth with complex root canal anatomies [19]. Further studies should be performed in the future, using a larger number of samples and different types of teeth in order to further investigate the impact of supplementary approaches on root canal disinfection.

The rDNA-based analysis showed that the inter-appointment medication did not contribute to an additional reduction of bacterial levels after CMP. Our data corroborate previous findings from rDNA-based molecular studies [23,24]. Moreover, a high prevalence of rDNA positive samples after inter-appointment medication has been previously reported [22,23,24,25]. An interesting finding of this study is that the rRNA-based analysis revealed the activity of bacteria detected by rDNA in S4 samples, proving it to be a useful strategy for monitoring the antibacterial effect of endodontic procedures. The inaccessibility of the bacteria present in the complex root canal system, the biofilm organization, and the intrinsic bacterial resistance to pH changes may have impaired the effect of Ca(OH)_2_ on the endodontic microbial community [30]. These findings confirm the need to search for new strategies to enhance root canal disinfection.

After the second root canal preparation, the number of rDNA-positive reactions in the S5 samples was significantly lower than in the S4 samples. Moreover, the lowest rDNA and rRNA levels were found at the end of the second treatment visit. Although previous culture-based microbiological studies have suggested that there is a bacterial reduction after second-visit instrumentation [29,31], they were unable to precisely assess these comparisons, due to the method’s low sensitivity and the possibility of false-negative results in samples taken after disinfection procedures. However, the sensitive molecular methods used in the present study highlighted a significant reduction in the bacterial load from root canals after second-visit instrumentation. Nevertheless, 30% of the canals remained positive for bacterial rDNA and rRNA at the end of the treatment. Considering that the bacterial DNA detected by qPCR at the time of the root canal filling may affect the treatment’s outcome [32], efforts are being made to recall the patients who participated in this study so that the correlation between the microbiological results and the success/failure of the treatment can be further investigated.

The use of both rDNA and rRNA (cDNA) as qPCR templates in the present study contributed to a better understanding of bacterial metabolism after endodontic procedures. However, rRNA-based assays are more laborious and costly than rDNA-based qPCR, which may limit their use in large sample studies. Moreover, the additional rRNA purification and cDNA synthesis steps prior to qPCR analysis make the process more time-consuming and prone to error. For instance, 5 out of 20 cases were lost during the reverse transcription reaction in the present study. Consequently, only 15 cases were available for the qPCR assay comparison. Although the frequency of bacteria detection tended to increase when using the rRNA-based assay, there was no significant difference between the two methods for assessing total bacteria in most samples. Moreover, all the rDNA-positive samples were also positive for rRNA when universal primers were used. In contrast, a difference in the methods’ sensitivity has been found in species-specific PCR assays, suggesting that there are differences in bacterial species susceptibility within the microbial community [33]. Ongoing studies are being conducted to investigate the susceptibility of different bacterial species in the samples studied, using rDNA- and rRNA-based qPCR assays.

In summary, this clinical study compared the antimicrobial effects of endodontic interventions. A treatment protocol that used XPF as a new tool for activating endodontic irrigants after CMP at the first treatment visit was compared with an inter-visit medication strategy. A pilot study was conducted to validate the clinical protocol for XPF activation, and a combined approach using XPF and PUI as adjunctive procedures was suggested. As expected, CMP promoted a drastic reduction in bacterial levels and activity. However, the tools used to activate endodontic irrigants did not contribute significantly to further disinfection after CMP. Bacteria were found to be active in root canals at the second visit, even after the use of an inter-appointment medication, but they were significantly reduced after a second-visit preparation. Consequently, the lowest bacterial levels were found at the end of the treatment. The present study, which used an rRNA and rDNA combined approach, confirmed the findings of previous studies using culture or DNA-based molecular analysis by showing that root canal preparation represents the main strategy for root canal disinfection. However, because one-third of the samples remained positive for both rRNA and rDNA at the end of the treatment, it is still necessary to search for new strategies to improve root canal disinfection.

## Figures and Tables

**Table 1:** Clinical characteristics of the 20 cases included in this study.

Clinical Features			
		*n*	%
Age	20–40	8	40
	>40	12	60
Gender	Male	9	45
	Female	11	55
Tooth type	Anterior	11	55
	Premolar	9	45
Root canal preparation	Reciproc instrument R40 (0.40 mm)	12	60
	Reciproc instrument R50 (0.50 mm)	8	40

**Table 2:** Bacterial rDNA detection by qPCR (number of positive samples) and median values (range) of rDNA levels in root canal samples taken after access cavity (S1), root canal preparation (S2), XP-endo Finisher (S3a), passive ultrasonic irrigation (S3b), calcium hydroxide medication (S4) and 2nd-visit root canal preparation (S5).

rDNA Levels	Root Canal Samples					
	S1	S2	S3a	S3b	S4	S5
Median (*n* = 20)	1.79 × 10^5 a^	7.58 × 10^3 b^	6.40 × 10^3 b^	3.82 × 10^3 b^	1.56 × 10^4 b^	0 ^c^
Range	(6.21 × 10^3^–2.52 × 10^6^)	(0–8.35 × 10^5^)	(0–7.37 × 10^5^)	(0–1.84 × 10^5^)	(0–2.09 × 10^5^)	(0–9.62 × 10^4^)
*n* (%)	20 (100%)	12 (60%)	11 (55%)	10 (50%)	14 (70%)	6 (30%)

rDNA levels: 16S rRNA genes quantified by qPCR (quantitative polymerase chain reaction). Different letters in horizontal lines (a, b, c) show significant differences in bacterial counts before and after root canal treatment procedures (nonparametric Wilcoxon signed rank test, *p* < 0.05). *n* = number of teeth included in the rDNA-based qPCR analysis; *n* (%) = number and percentage of samples with positive qPCR results.

**Table 3:** Bacteria detection rates by qPCR assays targeting bacterial ribosomal RNA (rRNA) and the respective genes (rDNA) in root canal samples taken after access cavity (S1), root canal preparation (S2), XP-endo Finisher (S3a), passive ultrasonic irrigation (S3b), calcium hydroxide medication (S4) and 2nd-visit root canal preparation (S5).

Samples			Detection Rates		*p*-Value *
			rDNA		
			+	−	
S1	rRNA	+	15	0	1.00
		−	0	0	
			+	−	
S2	rRNA	+	10	5	0.06
		−	0	0	
			+	−	
S3a	rRNA	+	9	4	0.12
		−	0	2	
			+	−	
S3b	rRNA	+	9	5	0.06
		−	0	1	
			+	−	
S4	rRNA	+	12	3	0.50
		−	0	0	
			+	−	
S5	rRNA	+	4	9	0.0039 *
		−	0	2	

* The McNemar test was used to compare the difference between the methods’ detection rates (*p* < 0.05 indicates significant differences). − and +, samples with negative and positive qPCR results, respectively.

**Table 4:** Median values (range) of rDNA and rRNA levels in root canal samples with qPCR positive reactions for both methods. Samples were taken after access cavity (S1), root canal preparation (S2), XPF (S3a), PUI (S3b), calcium hydroxide medication (S4), and 2nd-visit root canal preparation (S5).

	*n*	rDNA	rRNA	*p* Value *
S1	15	1.62 × 10^5^ (6.21 × 10^3^–2.52 × 10^6^)	4.08 × 10^5^ (6.27 × 10^4^–8.75 × 10^6^)	0.0007 *
S2	10	4.84 × 10^4^ (8.28 × 103–8.35 × 10^5^)	8.30 × 10^4^ (7.37 × 10^3^–6.80 × 10^5^)	0.5076
S3a	9	5.48 × 10^4^ (7.03 × 10^3^–7.37 × 10^5^)	5.50 × 10^4^ (1.06 × 10^4^–5.84 × 10^5^)	0.5940
S3b	9	4.48 × 10^4^ (7.63 × 10^3^–1.84 × 10^5^)	6.26 × 10^4^ (4.63 × 10^3^–3.66 × 10^5^)	0.2135
S4	12	7.58 × 10^4^ (7.40 × 10^3^–1.92 × 10^5^)	9.48 × 10^4^ (6.63 × 10^3^–4.41 × 10^5^)	0.0499 *
S5	4	8.91 × 10^3^ (4.63 × 10^3^–1.36 × 10^4^)	3.09 × 10^4^ (8.63 × 10^3^–5.97 × 10^4^)	N.A.

*n* = positive qPCR for both rDNA and rRNA. The symbol * indicates differences between median values of rDNA e rRNA in S1 and S4 samples (Wilcoxon signed rank test, *p* < 0.05). N.A.- not applicable.
